# Supplementary material for: Structural color three-dimensional printing by shrinking photonic crystals
Source: Nat Commun. 2019 Sep 25;10:4340. doi: 10.1038/s41467-019-12360-w (PMC6761189; doi:10.1038/s41467-019-12360-w)
Supplement: Supplementary file 1 — Supplementary Information [file 41467_2019_12360_MOESM1_ESM.pdf]

Supplementary Information

Structural Color Three-Dimensional Printing By Shrinking Photonic Crystals

Liu et al

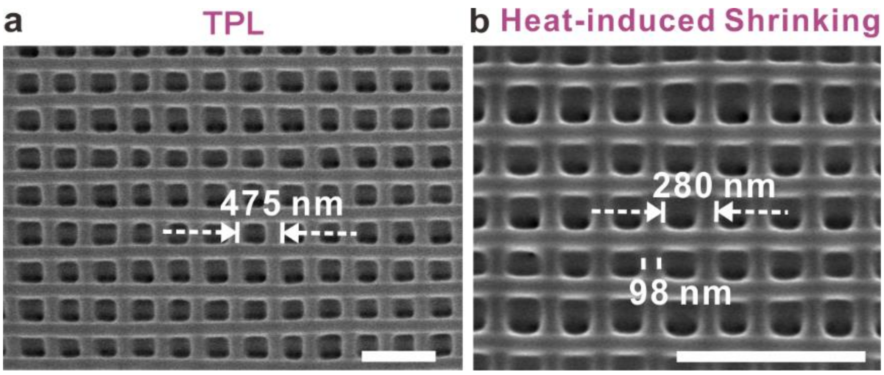

Supplementary Figure 1. Woodpile separate structures at the resolution limits of the conventional two-photon polymerization lithography (TPL) and heat-induced shrinking method. SEM images of (a) a woodpile with a_{xy} = 475 nm fabricated with TPL without heating and (b) a different woodpile with a_{xy} = 280 nm fabricated with the heat-induced shrinking method. The lattice constant of this woodpile prior to shrinking was 700 nm. Scale bars represent 1 μm. Parameters for patterning structure in (a): Write speed = 1 mm/s, laser power = 7.5 mW, nominal pitch 600 nm (note some shrinkage occurs after sample development). Parameters for patterning structure in (b): Write speed = 15 mm/s, laser power = 14.5 mW, nominal pitch preset = 700 nm.

Supplementary Note 1: Advantages and additional characterization of heat shrinking process

Supplementary Figures 2a and 2b compare woodpile structures fabricated by conventional two-photon polymerization lithography (TPL) and with heat induced shrinking. A key advantage of the heat shrinking process is the widening of the process window, enabling the fabrication of structures with smaller periods than is possible with TPL. Supplementary Figures 2c and 2d compare the volume fraction Φ of conventional TPL vs heat induced shrinking. Φ is calculated by:

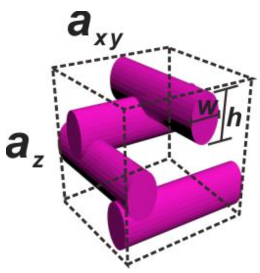

$$\Phi = 4\pi\left(\frac{w}{2}\right)\left(\frac{h}{2}\right)a_{xy}/a_{xy}^2 a_z$$

w: width of nanorod

h: height of nanorod

a_{xy}: lateral lattice constant

a_z: axial lattice constant

A Φ of 1 indicates unsuccessful fabrication where the structures have fused into a solid block. Our calculation ignores the effect of rod overlap and therefore gives a conservative estimate of the laser power and lattice constant where the structures fuse into a solid block. Woodpile photonic crystals with a_{xy} = 300 to 500 nm, a_z = 425 to 710 nm were fabricated with different laser powers. The smallest w and h of the constituent rods were obtained from SEM images, and Φ was calculated using a unit cell containing four rods with the arrangement shown in Supplementary Figure 2c. With conventional TPL, < 10% of the structures fabricated yielded $\Phi < 1$ with parameters $a_{xy} > 475$ nm and laser power < 17.5 mW. The smallest a_{xy} successfully fabricated was 475 nm with $\Phi = 0.9$ (Supplementary Figure 1a). Attempts at fabricating woodpiles with smaller lattice constants by using low laser power (7.5 mW) resulted in collapsed structures (Supplementary Figure 3c). With heat shrinking, well-defined and structurally sound woodpile photonic crystals with a_{xy} of 300 to 500 nm were successfully fabricated. Well-defined woodpile structures with a_{xy} ~280 nm and rod width ~100 nm were achieved (Supplementary Figure 1b).

In addition to decreasing the size and period of the 3D printed structure, heat shrinking also alters the effective shape of the writing spot, making it more spherical. As the Gaussian beam profile at the point of exposure has a depth of focus that is ~ 3x larger than the beam waist, the rod structures exposed with a single laser pass have elliptical cross-sections, as shown in Supplementary Figure 2a. After heat-induced shrinking, the cross-sections become more circular, as shown in Supplementary Figure 2b. The aspect ratio h/w for pre-heat shrunk structures is ~ 2.5 – 3.4, depending on the exposure power of the laser (Supplementary Figure 5). During the heat-induced shrinking process, there is a larger

decrease in height (39 – 60%) compared to the decrease in width (30 – 53%). The difference in shrinking is most pronounced in IP-Dip structures that have low degrees of cross-linking (exposed at low laser powers). With a laser power of 16 mW, the rods have h/w = ~210 nm / 105 nm = 2 after heat shrinking. The tendency of the structures to become more spherical suggests surface energy minimization during the shrinking process.

Other than woodpile photonic crystals, heat induced shrinking can also be used to fabricate other photonic crystal designs. To demonstrate this, we successfully fabricated 3D photonic crystals with gyroid[1] and diamond lattices[2,3] with periods of 375 and 290 nm, respectively (Supplementary Figures 2e and 2f). Such small feature sizes are comparable with previous reports of structures fabricated using stimulated emission depletion (STED).[4] Crucially, these structures could be achieved with higher throughput and/or lower laser powers as there is no competition between excitation and de-excitation processes here. Typically, it merely takes less than 30 s for printing a woodpile structure with dimension of 20 μm × 20 μm × 32 μm (a_{xy} = 1.5 μm, a_z = 2.1 μm).

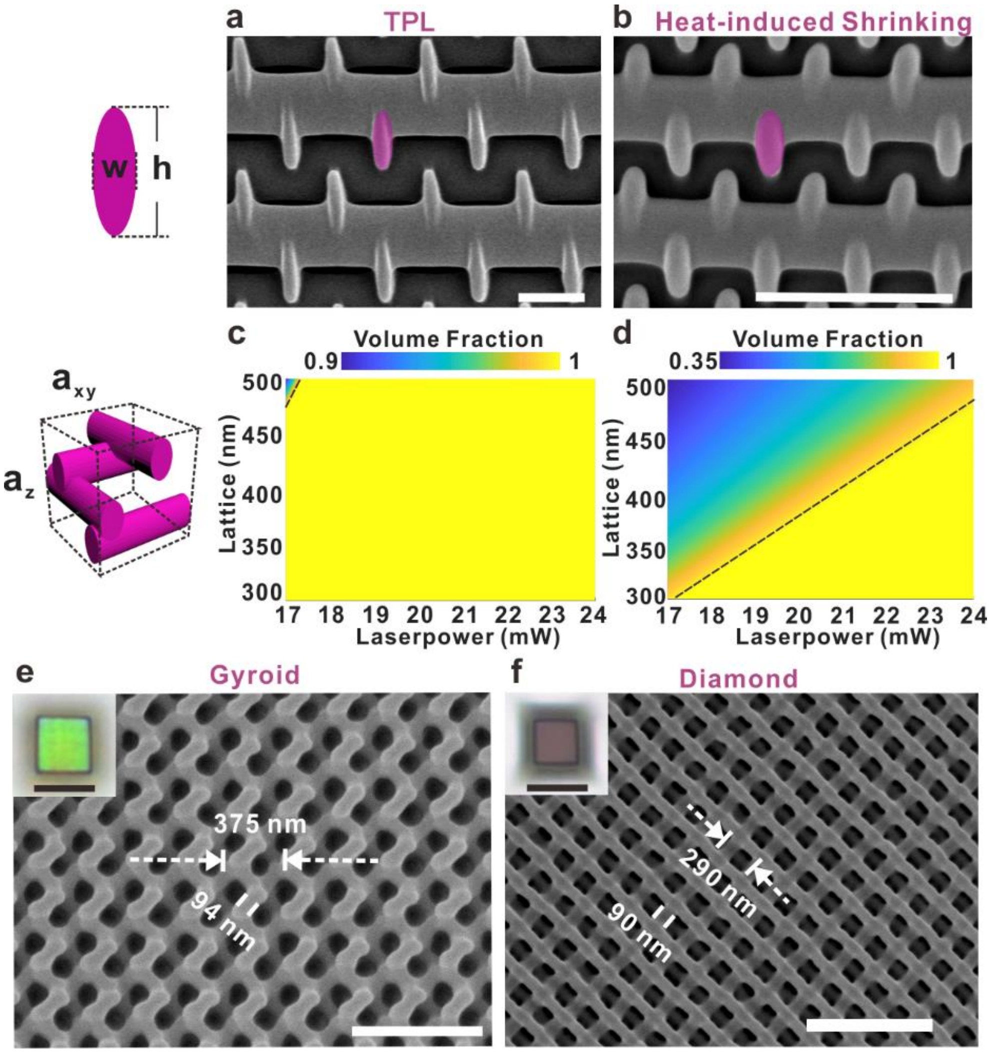

Supplementary Figure 2. The effect of heat shrinking on the effective writing spot shape, process window and the successful patterning of different photonic crystals. Side-view SEM images of woodpile photonic crystals fabricated by (a) two-photon polymerization lithography (TPL) with laser power of 20 mW and writing speed of 15 mm/s and (b) after heat treatment. Volume fraction maps of unit cells with lattice sizes less than 500 nm and made from various lithography laser powers, for (c) TPL and (d) our proposed heat treatment method, respectively. Optical micrographs (insets, scale bars represent 10 μm) and SEM images of (e) a gyroid photonic structure and (f) a diamond photonic crystal fabricated using the heat-induced shrinking method. Scale bars represent 1 μm.

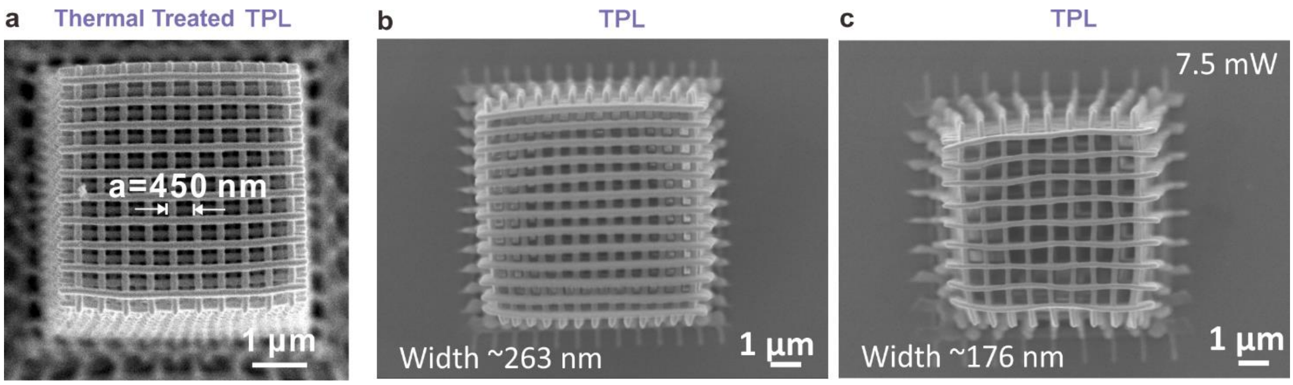

Supplementary Figure 3. Comparison of woodpile structures fabricated using conventional two-photon polymerization lithography (TPL) and with the heat-induced shrinking method. (a) With heat treatment, we were able to fabricate a structurally well-defined woodpile structure with a_{xy} = 450 nm (nearly the resolution limit of our Nanoscribe system) with well separated neighboring rods. (b, c) SEM images of woodpile structures with a_{xy} = 588 nm fabricated with conventional TPL. The rods were connected at various regions (b). This cannot be solved by adjusting exposure parameters, e.g. by lowering the laser power or write speed. Below the polymerization threshold of 7.5 mW, the IP-Dip was not sufficiently crosslinked and does not survive the development step, resulting in collapsed woodpile structures (c).

Supplementary Note 2: Estimated axial resolution (resolution in z-direction)

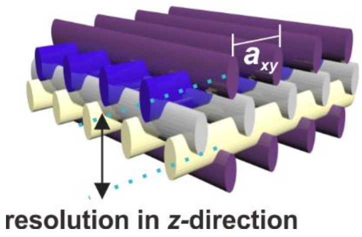

Supplementary Figure 4. Scheme showing axial resolution (R_z) in a woodpile structure. The axial resolution is described by the smallest center-to-center distance between two separated nanorods in z-direction, which is $3a_z/4$.

For lithographic woodpile structure before heating:

$a_z = \sqrt{2}a_{xy}$ (designed in lithography)

$R_z = 3\sqrt{2}a_{xy}/4$

When a_{xy} = 475 nm

$R_z = 475 \times 3\sqrt{2}/4 = 504$ nm

After heat-induced shrinking:

$a_z = S \times \sqrt{2} a_{xy}$, S is the factor of anisotropic shrinking in *xy*- and *z*-direction.

S = 1.89

$R_z = 1.89 \times 3\sqrt{2} a_{xy}/4$

When $a_{xy} = 280$ nm

$R_z = 380$ nm.

It is noteworthy that a good axial resolution is more challenging to achieve than a lateral resolution for TPL, as the axial Abbe limit is 2.92 times larger than the lateral Abbe limit.[4] With $a_{xy} = 475$ nm (smallest period that can be fabricated with our Nanoscribe system), the resolution limit in *z*-direction is estimated to be 504 nm (see calculation above). Using the heat-induced shrinking method, the smallest $a_{xy} = 280$ nm, corresponding to a resolution limit of 380 nm in the *z*-direction, which is below the two-photon Sparrow criterion in *z*-direction of 506 nm and comparable with the ultimate values achieved with STED ($a_{xy} = 275$ nm and *z*-resolution 375 nm).[4]

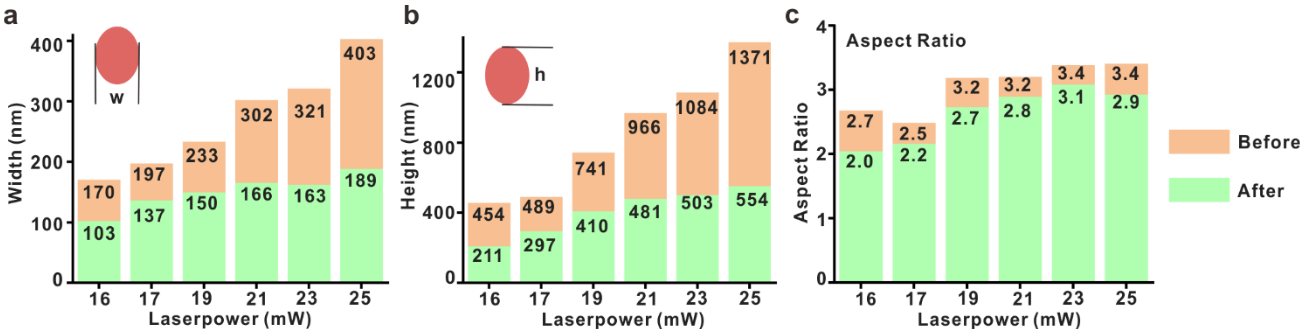

Supplementary Figure 5. Stack-bar charts showing dimensions of the effective writing spot before (orange) and after (green) heating for (a) the rod width, (b) the rod height and (c) the aspect ratio.

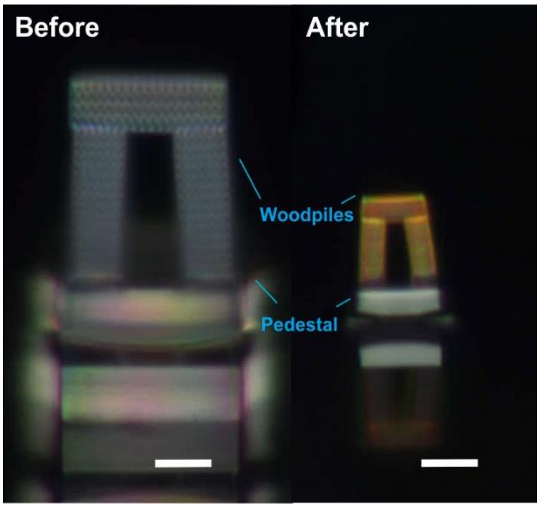

Supplementary Figure 6. Side-view images of photonic crystal structures sitting on pedestals made of fully cross-linked IP-Dip before and after thermal shrinking. All scale bars represent 10 µm.

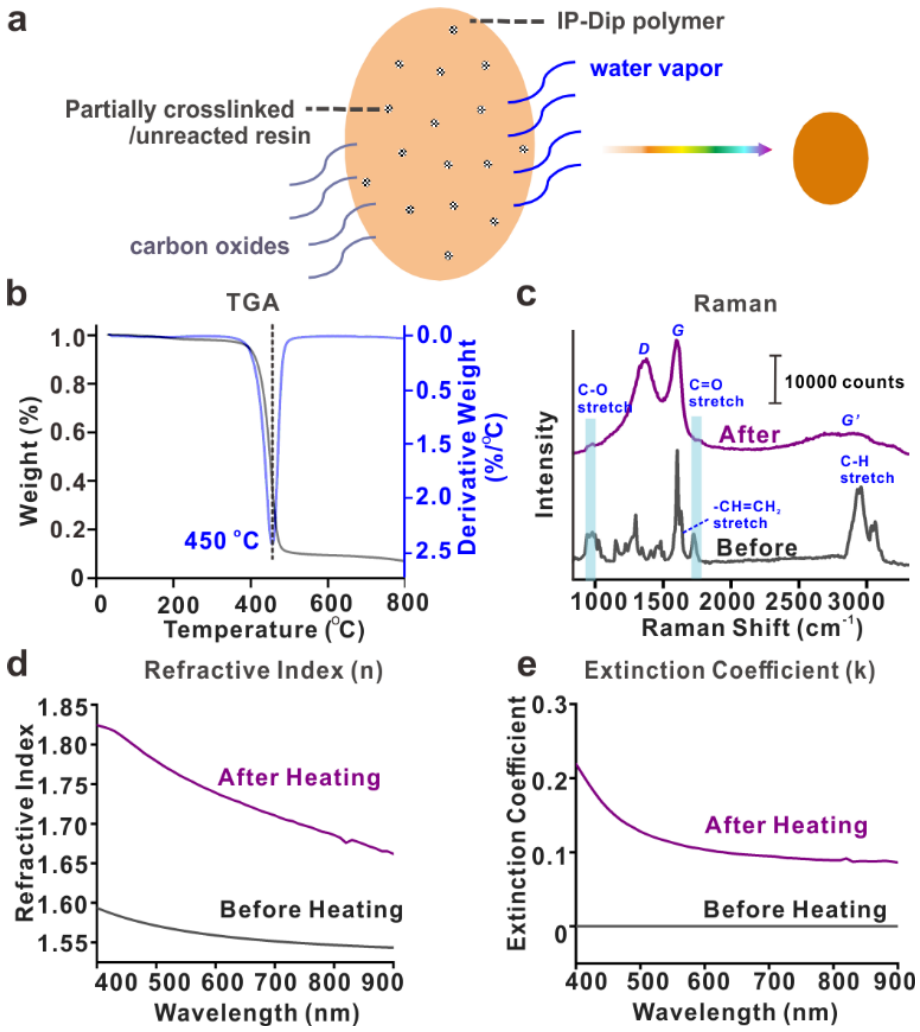

Supplementary Figure 7. Characterization of IP-Dip before and after thermal treatment. The main components of IP-Dip are 2-(hydroxymethyl)-2-[[(1-oxoallyl)oxy]methyl]-1,3-propanediyl diacrylate (CAS: 3524-68-3, 60-80%), 9H-fluorene-9,9-diylbis(4,1-phenyleneoxyethane-2,1-diyl)-bisacrylate (CAS: 161182-73-6, < 24%), and Biphenyl-2-ol, ethoxylated, esters with acrylic acid (CAS: 72009-86-0, < 24%) (a) Schematic showing the process by which the IP-Dip polymer loses volume, resulting in increased density and more carbon content. During the heating process, partially crosslinked resin (or unreacted resin) within the cured IP-Dip was removed. Pyrolysis of the polymer causes carbon oxides and water vapor to be released. (b) Thermogravimetric analysis of IP-Dip heated from 0 to 800 ºC. (c) Raman spectra of IP-Dip before and after heating. (d) Refractive indices and (e) extinction coefficients of IP-Dip before and after heating.

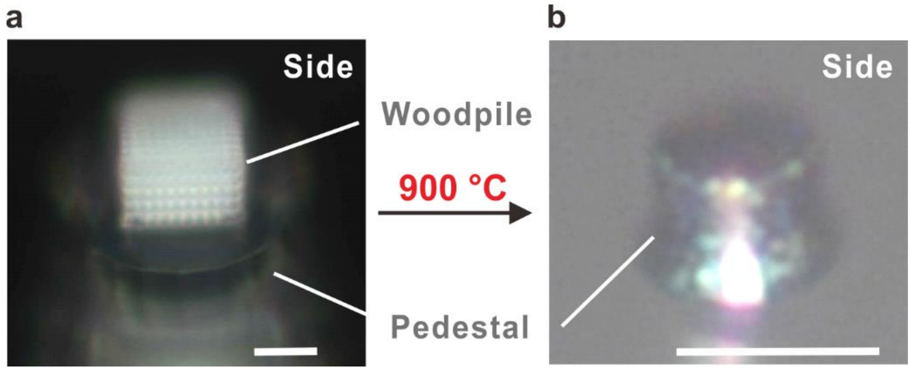

Supplementary Figure 8. Woodpile structure (a_{xy} = 1.9 µm, w = 403 nm) under pyrolysis at 900 °C. Side-view reflection images of the woodpile sitting on the pedestal made of fully cross-linked IP-Dip (a) before, and (b) after pyrolyzing at 900 °C. Woodpile did not survive in the pyrolysis and only the solid pedestal which is more structurally stable remained intact after heating to 900 °C. Scale bars represent 10 µm.

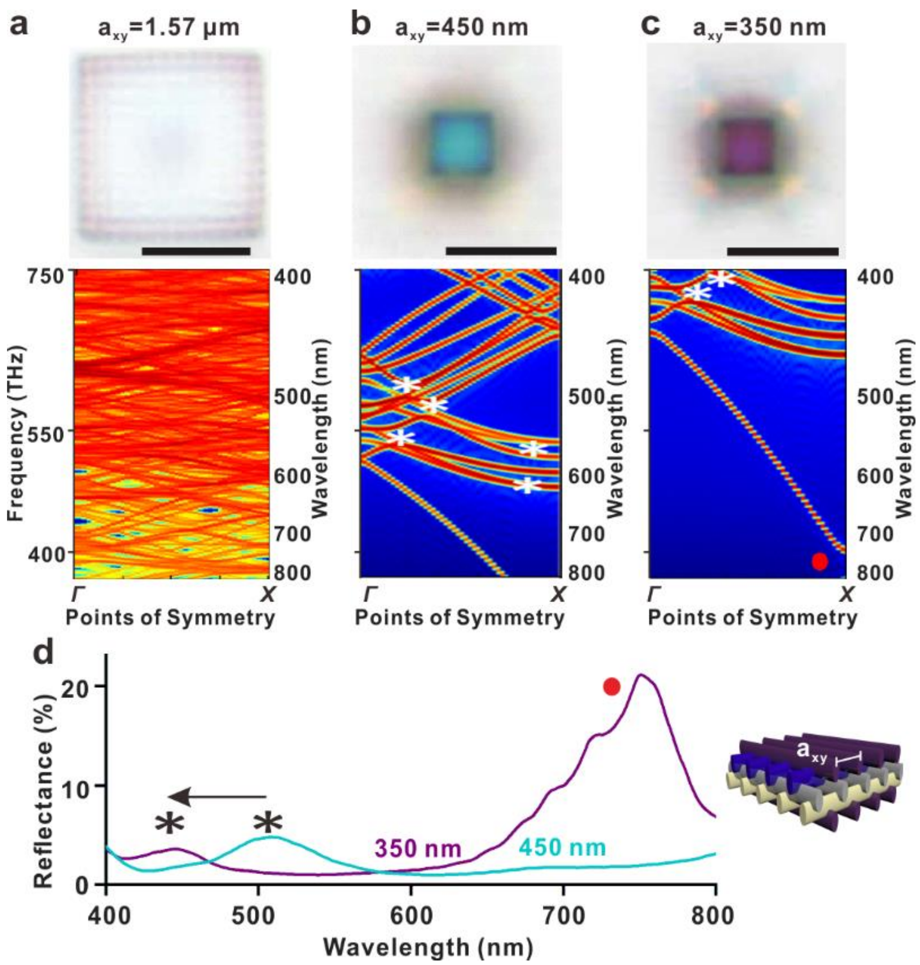

Supplementary Figure 9. Micrographs (top) and band structure diagrams (bottom) of woodpiles with (a) a_{xy} =1.57 µm before heating, and (b) a_{xy} = 450 nm and (c) a_{xy} = 350 nm. (d) Reflectance spectra for woodpiles with a_{xy} = 450 nm (blue) and a_{xy} = 350 nm (purple). Scale bars represent 10 µm.

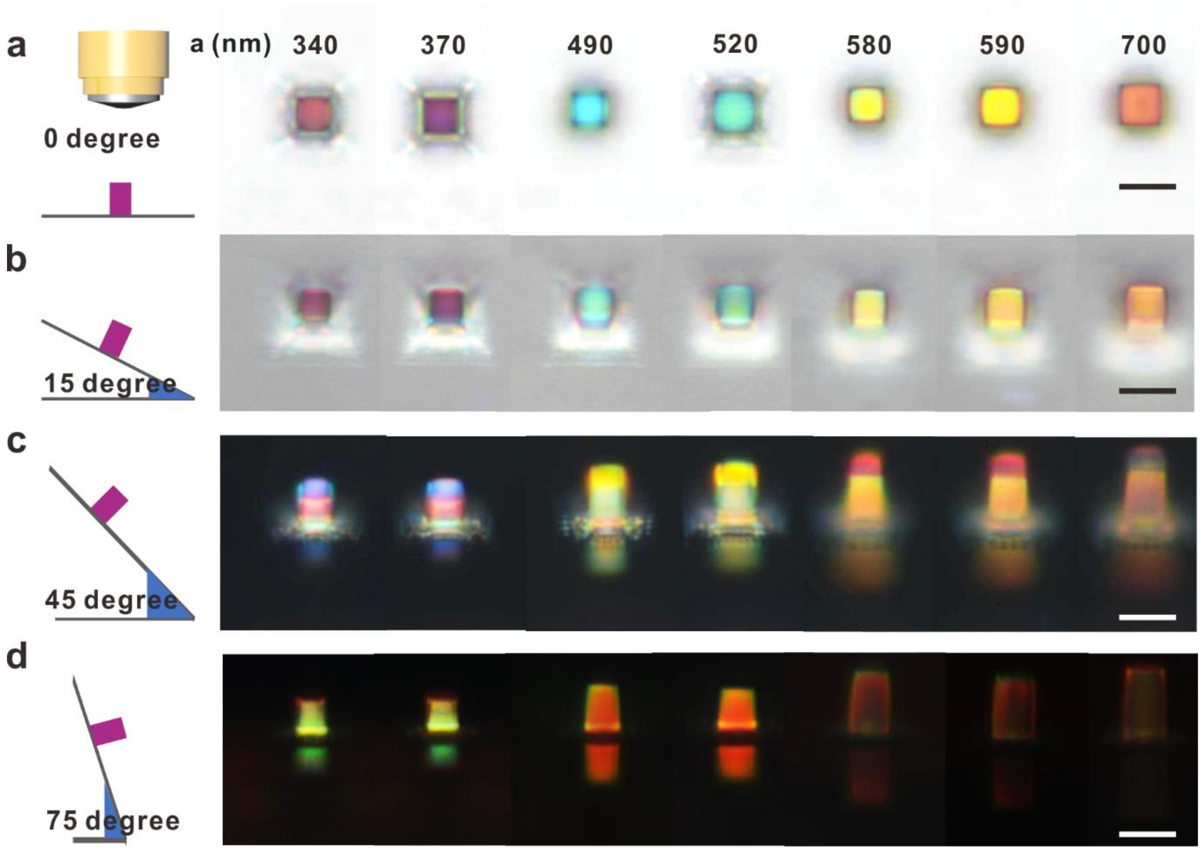

Supplementary Figure 10. Refection-mode optical microscope images showing angle-dependent color from woodpile structures with periods of 340-700 nm. The micrographs are taken at tilt angles of (a) 0°, (b) 15°, (c) 45° and (d) 75°. All scale bars represent 10 μm.

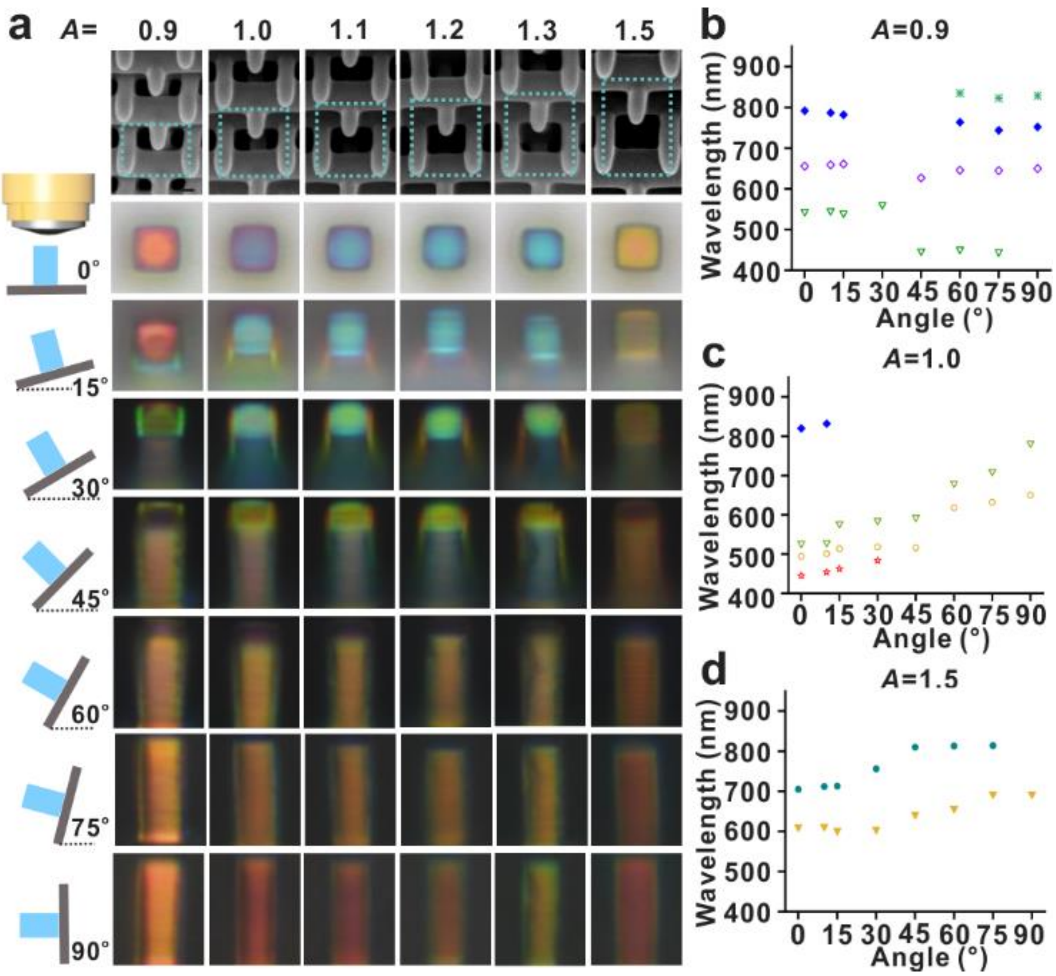

Supplementary Figure 11. (a) SEM (top panel) and reflection-mode micrographs of woodpiles fabricated with lateral period (a_{xy}) of ~450 nm and scaling factor A varying from 0.9 to 1.5, viewed at tilt angles of 0° to 90°. Plot of the peak wavelengths in the reflectance spectra measured at tilt angles 0° to 90° for (b) A=0.9, (c) A=1.0 and (d) A=1.5, respectively.

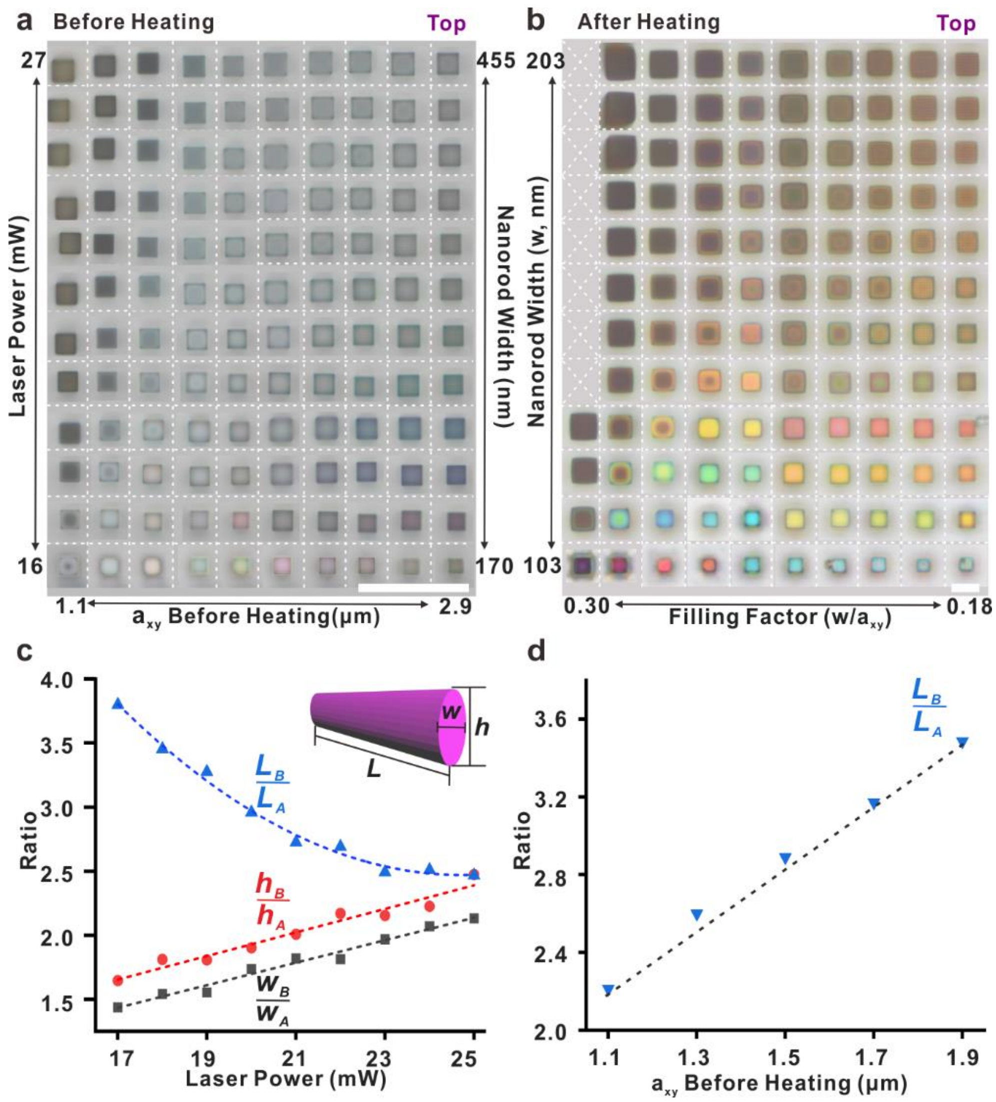

Supplementary Figure 12. 3D color voxels exhibiting a wide range of colors. (a) Top view reflection-mode micrographs of woodpile photonic crystals before heat shrinkage. a_{xy} varies from 1.1 to 2.9 µm across each row (step value 0.2 µm), and the laser power used during the writing process varies from 16 to 27 mW across each column (step value 1 mW). The rod width is a function of the laser power, and varies from 170 to 455 nm. (b) Top-view reflection-mode micrographs of the woodpile photonic crystals after heating. Heating the dense woodpiles produced with laser power from 20-27 mW induces their change into solid blocks and these solid structures are removed from B (in the first column). The lattice constant increases diagonally with colors shifting from magenta to blue, green, yellow, orange, and finally red. (c) Plot of L_B/L_A, h_B/h_A, and w_B/w_A for rods in woodpiles exposed using laser powers of 17-25 mW, where L_B (L_A), h_B (h_A), and w_B (w_A) denote the length, height and width of the rods before

(after) heating, respectively. The dotted lines are fitting curves. (d) Plot of L_B/L_A for rods fabricated with two-photon polymerization lithography (without heat shrinkage), with a_{xy} varying from 1.1 to 1.9 µm. The scale bars in (a) and (b) represent 100 µm and 10 µm, respectively.

Supplementary Note 3: Different Shrinkage Rates

The manner in which the woodpile structures shrink was investigated and plotted in Supplementary Figures 13c and 13d. The shrinkage was characterized by the ratio of each dimension measured from SEM images before and after heating, i.e. w_B/w_A, h_B/h_A *and* L_B/L_A. We observe that with increasing laser power, i.e. higher degree of crosslinking, the rod shrinkage decreased along the length of the rod but increased along the shorter axes, i.e. *h* and *w*. Furthermore, for a given laser power, the degree of shrinkage increases with increasing period. These results show intuitively that the largest shrinkage is achieved for sparse, weakly crosslinked structures.

The heat-induced shrinkage is anisotropic in width (*w*), height (*h*) and length (*L*) of nanorods in woodpile structures. At certain exposure laser power, the shrinkage follows $w_B/w_A < h_B/h_A < L_B/L_A$ to reduce the surface energy, indicating that the lattice constant in *xy*-direction reduces more than in *z*-direction for woodpile structures (Supplementary Figure 12c). In this case, the geometry of the unit cell is elongated along z after heating and the factor of elongation (*E*) is a function of laser exposure power (Supplementary Figure 13a). In order to fabricate woodpile unit cells with a certain geometry (a_z/a_{xy}), a compensating factor (*C*) can be multiplied to a_z in design such that $C = 1/E$ (Supplementary Figure 13b).

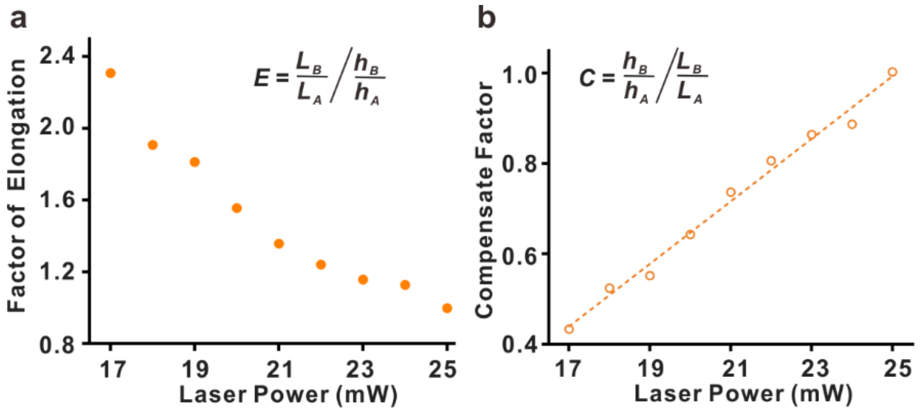

Supplementary Figure 13. Plot of (a) factor of elongation (*E*) and (b) compensation factor for anisotropic shrinkage in *xy*- and *z*-direction, as a function of laser power.

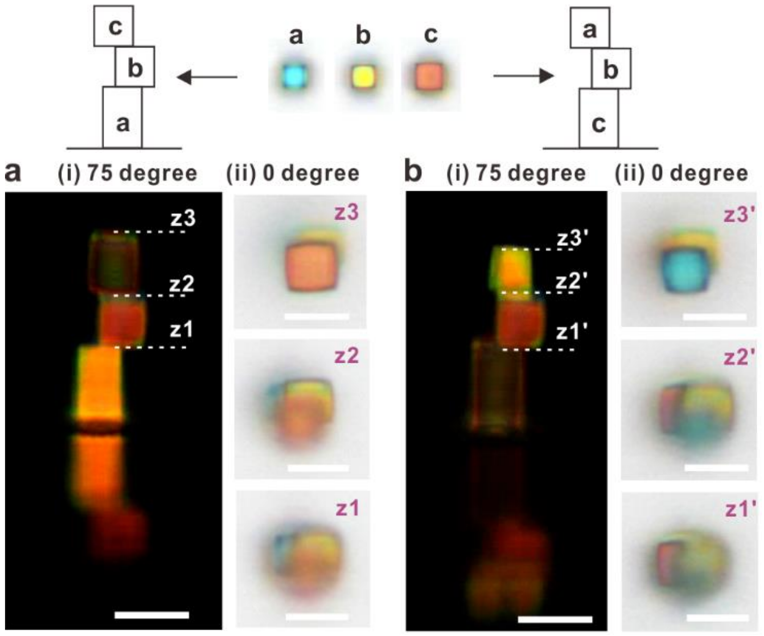

Supplementary Figure 14. Colored "ladder" (3D stacked cubes) demonstrating a simple 3D printed color object. (a, b) Reflection-mode optical micrographs of two 3D color voxels stacks. *a*, *b* and *c* are woodpile voxels with different periods. Voxel *a* is placed at the bottom in (a) and at the top in (b). Reflection-mode optical micrographs are taken at tilt angles of (i) 75 degree and (ii) 0 degree, and optical micrographs images are taken at three focal planes in (ii). All scale bars represent 10 µm.

We constructed two "ladder" structures (3D stacked cubes) to demonstrate the ability to print 3D color objects and its multiplexing capability. These two ladders both comprise of three color voxels (a, b and c) with different stacking orders. Voxels a, b and c were chosen from the library of shrunken

woodpiles exhibiting cyan, yellow and orange colors at normal incidence (0 degree). Their periods are 500, 580 and 700 nm, respectively. After thermal shrinking, the ladder structures exhibit vivid colors, with different colors appearing at different focal planes and viewing angles. Reflection-mode optical micrographs demonstrate that the colors of the three voxels could be read out independently at both viewing angles. The shrinking percentage and the resultant color were almost identical for woodpile structures with the same lattice constant in the two stacks, and the shrinking can therefore be considered to be independent of neighboring voxels. As such, we could arbitrarily put the color voxels at the top or at the bottom and individually read out them via reflectance colors observed from side, indicating the multiplexing capability of our strategy.

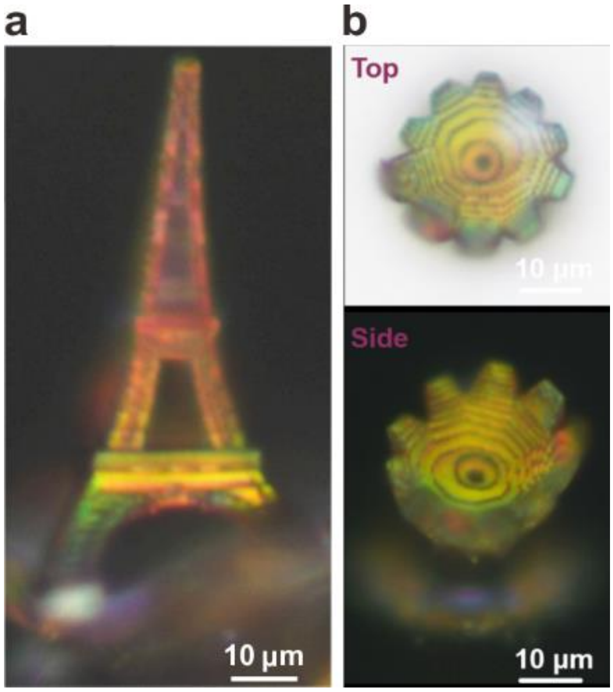

Supplementary Figure 15. Full-color models printed with heat induced shrinking method. Optical micrographs of models of the (a) Eiffel Tower and (b) ArtScience Museum in Singapore.

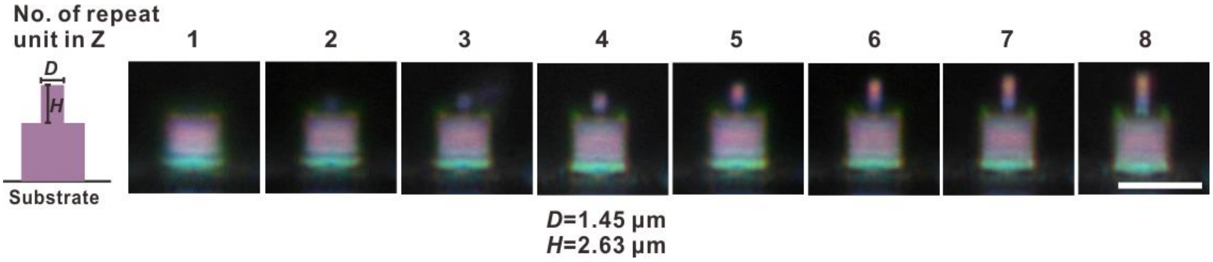

Supplementary Figure 16. Reflection-mode micrographs for voxel height testing. On top of the sacrificial pedestal, thin pillars made of woodpile structures are fabricated at a fixed D of 1.45 μm which is the smallest achievable. The height of the pillars is increased from left to right by increasing the number of repeat units in the z-direction, from 1 to 8. We observe from the micrographs that color can be seen only when the number of repeating units is more than 4, where the height is 2.63 μm. This represents the smallest voxel that we can fabricate. Scale bar represents 10 μm.

Supplementary References

1 Gan, Z., Turner, M. D., Gu, M. Biomimetic gyroid nanostructures exceeding their natural origins. *Science Advances* **2**, e1600084 (2016).

2 Castles, F. *et al.* Microwave dielectric characterisation of 3D-printed BaTiO 3/ABS polymer composites. *Scientific Reports* **6**, 22714 (2016).

3 Maldovan, M., Thomas, E. L. Diamond-structured photonic crystals. *Nature Materials* **3**, 593-600 (2004).

4 Fischer, J., Wegener, M. Three-dimensional optical laser lithography beyond the diffraction limit. *Laser & Photonics Reviews* **7**, 22-44 (2013).
